# Measuring leprosy case detection delay and associated factors in Indonesia: a community-based study

**DOI:** 10.1186/s12879-023-08552-x

**Published:** 2023-08-25

**Authors:** Yudhy Dharmawan, Ida J. Korfage, Ulfah Abqari, Bagoes Widjanarko, Jan Hendrik Richardus

**Affiliations:** 1 Department of Public Health, Erasmus MC, University Medical Center Rotterdam, Rotterdam, the Netherlands; 2 Faculty of Public Health, Universitas Diponegoro, Semarang, Indonesia; 3 NLR Indonesia, Jakarta, Indonesia

**Keywords:** Leprosy, Disability, Case detection delay, Indonesia

## Abstract

**Background:**

Leprosy is a public health burden in Indonesia with a high number of new cases every year and a high proportion of disability among new cases. Case detection delay (CDD) can contribute to ongoing transmission and increased disability chances among leprosy patients. This study aimed to establish the CDD of leprosy and the factors associated with detection delay in Indonesia.

**Method:**

Community-based study with a cross-sectional design. Data were collected through interviews about sociodemographic and behavioral factors, anticipated stigma, and duration of CDD. Leprosy classification and case detection methods were obtained from health service records. A random sample was taken of 126 leprosy patients registered between 1st October 2020 and 31st March 2022 in the Tegal regency in the Central Java Province. Data were analysed by descriptive and analytical statistics using multiple linear regression.

**Results:**

The mean CDD, patient delay, and health system delay were 13.0 months, 9.7 months, and 3.2 months, respectively. Factors associated with longer CDD are younger age (below 35 years), male, found through passive case detection, and not having a family member with leprosy. Factors associated with longer patient delay were being younger (below 35 years), being male, not having a family member with leprosy, and anticipated stigma of leprosy. It was not possible to reliably identify factors associated with health system delay.

**Conclusion:**

CDD in leprosy should be reduced in Indonesia. The Indonesian National Leprosy Control Program (NLCP) is advised to adopt an integrated intervention programme combining active case detection with targeted health education to reduce CDD and thereby preventing disabilities in people affected by leprosy.

**Supplementary Information:**

The online version contains supplementary material available at 10.1186/s12879-023-08552-x.

## Background

Globally annual new case detection fell from around 750,000 in 2000 to just over 200,000 in 2019 [1]. Leprosy remains a public health problem because of the physical disability and social stigma it causes. Therefore, the WHO global target for leprosy is a 70% reduction in the annual number of new cases detected and a 90% reduction in the rate per million population of new cases with Leprosy Grade 2 Disability (G2D) as compared to baseline numbers reported in 2020 [2].

In 2000, the prevalence rate of leprosy was 0.9/10,000 population in Indonesia, and thereby leprosy was eliminated as a public health problem (which is defined as a prevalence rate of less than 1/10,000 population) [3]. Nevertheless, Indonesia has the third-highest number of new leprosy cases and leprosy cases with disability (indicated as G2D) in the world, after Brazil and India [1]. G2D in leprosy is defined as visible deformities due to leprosy neuropathy [4]. G2D has been proposed as a more appropriate and robust indicator for disease burden than leprosy prevalence because it is less susceptible to operational factors such as detection delay [5]. Indirectly, G2D also provides information on other factors that influence case detection, such as community awareness about leprosy, the capacity of health staff to recognise early signs and symptoms, and, to some extent, the quality of the leprosy health services themselves [6]. Between 2001 and 2019, the incidence of leprosy in Indonesia remained stable, with the number of newly diagnosed leprosy cases ranging between 17,000 and 20,000 per year [7]. According to the Indonesian National Leprosy Control Program (NLCP), the number of new cases of leprosy in 2021 was 10,976 and the prevalence rate was 0.43 per 10,000 population [3, 8, 9]. These lower figures are probably due to disruption of the leprosy case detection programme because of the Covid-19 pandemic, as was also the case in many other leprosy control programmes worldwide [9, 10]. The rate of G2D in 2000 was 6/1,000,000 population, increased to 8.7/1,000,000 in 2011, and decreased again to 2.47/1,000,000 in 2021, although this figure probably does not reflect the real situation due to the disruption in the leprosy programme caused by the Covid-19 pandemic [3, 8, 9]. The G2D rate in Indonesia is more than 1/1,000,000 and thereby considered by the WHO represent a high leprosy burden [9, 11, 12].

The number of new leprosy cases with G2D is an indicator of delayed detection. The reduction of detection delay is crucial for two reasons: (1) to reduce the transmission of *Mycobacterium leprae*, the causative agent of leprosy, and thereby reducing the number of new leprosy cases annually, and (2) to reduce the number of new leprosy cases with G2D and thus preventing disability in leprosy patients. Delayed case detection is associated with disability in leprosy because nerve damage usually becomes irreversibly after six months of onset [13]. The diagnosis should ideally be made within six months, allowing treatment with corticosteroids to reduce inflammation in and around peripheral nerve fibers caused by leprosy and thereby preventing loss of nerve function and the resulting disabilities [14]. Early detection and treatment before disabilities develop remains the key strategy in leprosy to halt transmission of leprosy in the community and to prevent disability [4]. Since passive case detection is ineffective to improve early case detection, active case detection is encouraged [15]. Early case detection should therefore be a priority in any leprosy control programme [2].

To develop an efficient and effective intervention to improve early case detection of leprosy in Indonesia, it is necessary to know the factors related to case detection delay (CDD). Currently, the factors related to leprosy CDD in Indonesia are not fully known. Also, there is no clear information about the duration of CDD of leprosy in Indonesia [16]. This study aimed to establish CDD of leprosy in Indonesia and the associated factors. The results of this study can lead to recommendations to improve early case detection of leprosy and thereby reducing the transmission of *M. leprae* and the occurrence of G2D in leprosy patients.

## Methods

### Study Design

This community-based cross-sectional study established the duration of the CDD of leprosy in months and identified factors associated with this CDD. It was conducted according to the STROBE Statement (Additional file 1: Table S1) [17]. Data were collected through questionnaires that were completed during interviews by trained interviewers with patients who were diagnosed with leprosy. Sociodemographic factors included age, sex, years of education, living area, marital status, employment, and the distance from home to the healthcare service. Behavioral factors included knowledge about leprosy, the first action taken by people with leprosy to seek medication, the first healthcare service visited, and the number of examinations by or visits to healthcare services before leprosy was diagnosed. Furthermore, anticipated stigma was studied (see below). Clinical data from health records were used to identify the method of case detection of leprosy. We distinguished passive case detection if patients visited healthcare services, and active case detection if patients were found through case detection activities in the community. Leprosy was classification as either paucibacillary (PB) or multibacillary (MB) leprosy, according to the World Health Organization (WHO) leprosy guidelines, which are also used by the Indonesian National Leprosy Control Program (NLCP) [7].

### Study area

The study was carried out in June 2022 in Tegal Regency, Central Java Province, which is a leprosy endemic area on Java Island in Indonesia. Java Island has the largest population and the highest number of leprosy cases in Indonesia [9]. Tegal Regency attained elimination as public health problem (prevalence < 1/10,000 population) in 2021 when the prevalence rate reached 0.8/10,000 population. The lower prevalence rates in 2020 and 2021 are probably due to the disruption of the leprosy case detection programme because of the Covid-19 pandemic, as was also the case in Indonesia and globally. Although the prevalence rate had decreased since 2017, the percentage of G2D in new cases remained around 10% [18, 19]. The leprosy prevalence rate and G2D percentage in new cases in Tegal Regency are higher than in Indonesia overall [9, 19]. The higher cases and G2D percentage of leprosy patients indicate that leprosy is still a public health burden in the Tegal Regency [11, 20].

### Population and Sample

**Fig. 1:**
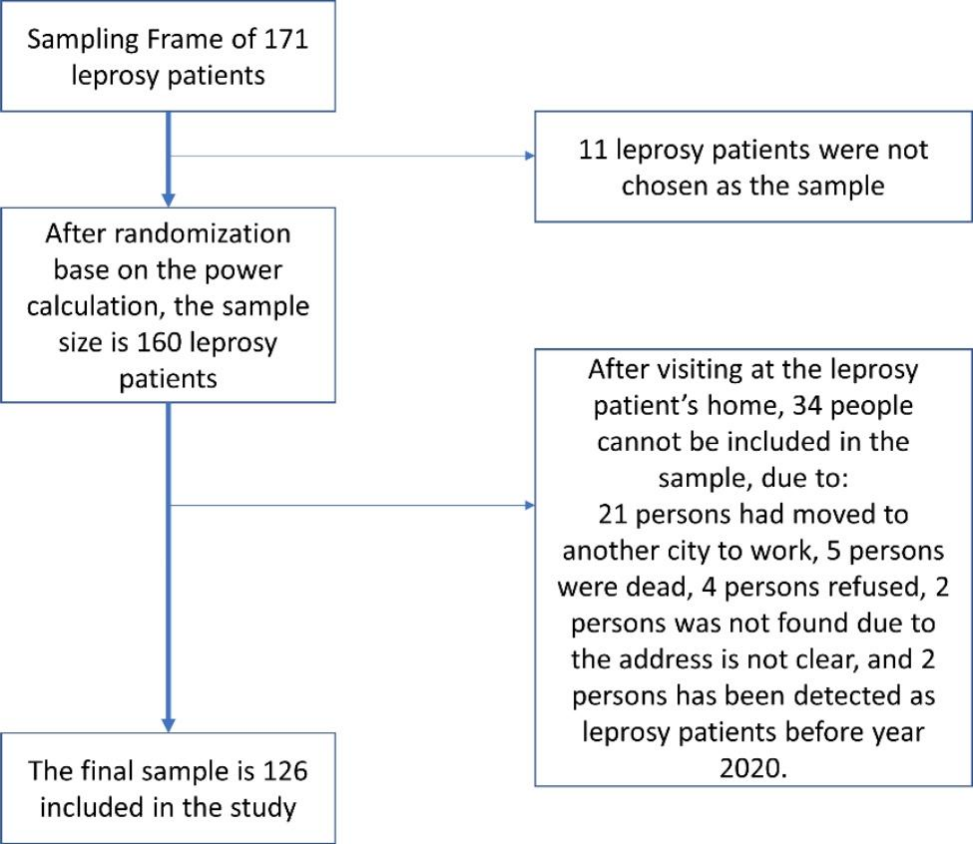
Flow diagram of sample selection

The study population included people affected by leprosy living in Tegal Regency. The target population consisted of people registered at the District Health Office of Tegal Regency between 1st October 2020 and 31st March 2022 as having leprosy. We assumed that having been registered recently would enhance people’s ability to remember the duration of CDD and thereby minimising recall bias. We applied the following inclusion criteria: receiving treatment for leprosy or being released from leprosy treatment, being able to provide informed consent, and being able to communicate in Bahasa Indonesia [21]. The required sample size was calculated using the formula for cross-sectional studies, with an estimated proportion of 10% of patients having G2D [22]. We thus derived a sample size of 139 (CI 95%, d 5%). Assuming a non-response rate of 10%, the final sample size was 153, rounded to 160 respondents. By means of computerised random sampling we selected 160 patients from the sampling frame of 171 patients aged 18 to 65 years (Fig. 1). Finally, we were able to recruit 126 respondents.

### Measurement tools

The interview contained questions about socioeconomic, community, and demographic factors, formulated on the basis of research experience in Indonesia [21]. Regarding anticipated stigma of leprosy in the community, we first asked the respondents one question to ensure that they had heard of leprosy. We then asked three questions based on the SARI STIGMA scale [21]. These questions were: (1) Did you expect that other people would think differently about you if they knew about the sign on your body? (2) If yes, why do you expect they would think differently about you? (3) Did you talk with anybody about the disease after it was diagnosed? (Additional file 2: Text S1). The scale of the anticipated stigma ranges from 0 to 8, with higher scores indicating more anticipated stigma.

To measure knowledge about leprosy, we used the eight knowledge questions from the KAP (Knowledge, Attitude and Practice) Questionnaire that had been developed for the Indonesian context [23, 24]. The score was calculated by adding up the correct answers, even if incorrect answers were present. The knowledge score ranges from 0 to 9. We define ‘poor knowledge’ as a score of 3 or less (< 33% correct), ‘moderate knowledge’ as a score between 3 and 6 (33–67% correct), and ‘adequate knowledge’ as a score of 6 or more (> 67% correct) [23, 24]. The questionnaire can be found online. (https://www.leprosy-information.org/toolkits/perception-study-toolkit-pst-0).

We measured CDD as the duration from the moment that the respondent noticed the first symptom or sign of leprosy until the leprosy diagnosis. The duration was in months using the CDD questionnaire that has been validated in the Indonesian context [25]. We described patient delay as the duration from onset of the symptom or sign until the first visit to a healthcare service. Health system delay was defined as the period from the first visit to a healthcare service until receiving the leprosy diagnosis [26, 27]. We categorised a CDD period with a threshold of 6 months in which more than 6 months means that lasting nerve damage and the resulting disabilities are more likely to occur [14]. The CDD questionnaire also contained questions about health seeking behavior of leprosy patients from noticing the initial signs of leprosy until having leprosy diagnosed by the appropriate healthcare services [25, 28]. The questionnaire can be found at https://www.leprosy-information.org/resource/case-detection-delay-questionnaire [28].

### Data Collection and Analysis

Data collection took place through interviews by trained Indonesian interviewers with appropriate academic background. The interviewers also collected clinical data of leprosy patients recorded in the patient chart at the healthcare service. With the permission of healthcare staff, interviewers visited eligible leprosy patients. The interviewers introduced themselves as healthcare staff partners, explained the study, and asked for their informed consent.

Data were entered into an SPSS database and analysed using descriptive and analytical analyses. Means (with 95% CI) and medians were used to summarise numerical data, including the main outcome measure (CDD). Frequency distributions and percentages were presented for categorical data, including the duration of CDD for each group. We used multivariable linear regression analysis to investigate factors related to CDD [29]. First, the correlation between the independent variables with CDD as the dependent variable was tested with the Pearson correlation test. The data on CDD were transformed into a log distribution [30]. Factors associated with CDD were identified through backward selection in a model containing all candidate factors [31]. Factors with the highest p-value were dropped from the model until all factors had a p-value of < 0.05.

To explain the variation of the multiple regression model factors related to CDD, we also calculated the determinant coefficient (R^2^). The underlying assumptions of the linear regression regarding linearity, homoscedasticity, and normality and independency of the residuals were assessed graphically. Multicollinearity between candidate factors was assessed using the Variance Inflation Factor (VIF) [32–35]. Quantitative analysis was conducted using SPSS 27.

### Ethical considerations

This study was carried out according to the principles stated in the Declaration of Helsinki and followed The International Ethical Guidelines for Health-related Research Involving Humans prepared by the Council for International Organizations of Medical Sciences (CIOMS) in collaboration with the World Health Organization (WHO) 2016.

#### Ethical approval

was obtained from the Faculty of Public Health, Universitas Diponegoro Semarang in Indonesia, with certificate number 43/EA/KEPK-FKM/2022. All study respondents were given study information with details about leprosy, the study purpose, the right to withdraw, and the confidentiality of the disease status of the respondent. All respondents gave written informed consent [36].

## Results

### Characteristics of respondents

Table 1 provide information on the factors related to CDD of the 126 respondents in the study. Seventy-nine respondents (63%) were male. The age of the respondents age ranged from 18 to 65 years, with a mean of 42.0 years (95% CI: 39.9–44.1). Fifty-nine (47%) respondents were in the age group of 35–50 years. Of all, 100 (75%) had completed primary or secondary education, and the mean number of years of education was 7.5 years (95% CI: 6.8–8.2). Eighty-five (68%) respondents were employed, 91 (72%) were married, and 87 (69%) lived in a rural area. The mean distance from home to the nearest healthcare center was 3.75 km (95% CI: 3.2–4.4), with 107 (85%) respondents living in areas with a distance to a healthcare center of less than 5 km. One hundred and two respondents (81%) had MB leprosy, and 80 (64%) were diagnosed through passive case finding.

### Case detection delay

The mean CDD was 13.0 months (95% CI: 10.3–15.6), with a median of 5.5 months. The mean patient delay was 9.7 months (95% CI: 7.3–12.2), with a median of 3.0 months. The mean health system delay was 3.2 months (95% CI: 2.0–4.5), with a median of 0 months. Nearly half (60, 48%) of the respondents had a delay of more than six months.

The mean CDD was longer in the age group 18–34 years (x̅ =20.0; 95% CI: 13.6–26.3) than in the age groups of 35–50 years (x̅ =12.1; 95% CI: 8.4–15.8) and 51–65 years (x̅ =6.5; 95% CI: 3.8–9.2). The delay mean was also higher in males (x̅ =14.4; 95% CI: 10.9–17.9) than in females (x̅ =10.5; 95% CI: 6.4–14.7). Furthermore, the mean delay was higher in employed people (x̅ =13.5; 95% CI: 10.2–16.8) than in those who did not work (x̅ =11.9; 95% CI: 7.3–16.6). Respondents living in urban areas (x̅ =16.0; 95% CI: 9.7–22.4) had a longer delay than those living in rural areas (x̅ =11.6; 95% CI: 8.9–14.3) (Table 1).

The CDD (Table 1) was longer for respondents with MB leprosy (x̅=13.2; 95% CI: 10.3–16.0) than for PB leprosy (x̅=13.1; 95% CI: 10.3–16.0). Respondents found through passive case detection had a longer CDD (x̅=16.5; 95% CI: 12.8–20.1) than patients found by active case detection (x̅=6.7; 95% CI: 3.9–9.9).

**Table 1:** Sociodemographic characteristics of respondents and factors of case detection delay

Variables		Frequency	%	Mean CDD	95% CI
a. Sociodemographic characteristics					
Age groups	18–34 years	36	29	20.0	13.6–26.3
	35–50 years	59	47	12.1	8.4–15.8
	51–65 years	31	24	6.5	3.8–9.2
Sex	Male	79	63	14.4	10.9–17.9
	Female	47	37	10.5	6.4–14.7
Education Groups	No School or incomplete elementary school	26	21	7.1	3.8–10.4
	Complete education at primary school or a higher level of education	100	79	14.5	11.3–17.7
Occupational	Non-Employed (Does not have a job)	41	32	11.9	7.3–16.6
	Employed	85	68	13.5	10.2–16.8
Marital Status	Unmarried	35	28	14.0	8.3–19.8
	Married	91	72	12.6	9.6–15.6
Area of Residence	Rural	87	69	11.6	8.9–14.3
	Urban	39	31	16.0	9.7–22.4
Distance	5 km or less	107	85	12.3	9.6–15.0
	More than 5 km	19	15	16.8	7.0-26.6
b. Clinical factors					
Leprosy Type	PB	24	19	12.3	4.7–19.8
	MB	102	81	13.1	10.3–16.0
Case detection method	Passive	80	64	16.5	12.8–20.1
	Active	46	36	6.9	3.9–9.9
c. Behavioral factors					
Knowledge	Poor^a^	10	8	8.2	1.8–14.6
	Moderate^b^	44	35	13.0	8.7–17.3
	Adequate^c^	72	57	13.6	9.8–17.5
Healthcare-seeking behavior (First action)	Initially ignored the leprosy symptoms	21	17	9.9	3.5–16.3
	Self-medication and seeking not appropriate medicine and healthcare services	57	45	13.1	9.6–16.7
	Visiting appropriate healthcare service	48	38	14.1	9.0-19.2
First healthcare visited	Medical Doctor (GP)	36	29	12.4	7.9–17.0
	Neurologist	1	1	0	It cannot be analysed
	Hospital	6	5	23.3	-8.0-54.7
	Health center	67	53	12.1	8.6–15.6
	Dermatologist	16	12	14.9	7.3–22.5
Number of consultations/visits to healthcare before leprosy diagnosis	0	50	40	10.0	6.4–13.6
	1	36	29	17.8	11.3–24.4
	2	17	13	13.5	5.6–21.5
	3 or more	23	18	11.4	6.6–16.2
d. Anticipated stigma					
Having family member with leprosy	No	100	79	14.5	11.3–17.6
	Yes	26	21	7.2	3.0-11.4
Heard about leprosy before being diagnosed with leprosy	No	72	57	13.4	9.5–17.2
	Yes	54	43	12.5	8.8–16.1
Expectation that other people would think differently about them if they knew about the sign on their body	No, not at all	78	62	12.2	8.8–15.6
	Yes, maybe a little bit	23	18	15.3	9.4–21.2
	Yes, absolutely	25	20	13.2	6.2–22.1
Expectation that other people would think differently about them because a leprosy patient is considered unclean (*kush*)	No	122	97	12.8	10.1–15.5
	Yes	4	3	18.5	7.1–29.9
Talked with anybody about the disease after being diagnosed with leprosy	No	6	5	22.0	2.3–41.7
	Yes, only with family or close friends	111	88	12.0	9.5–14.6
	Yes, with anybody who showed interest	9	7	18.8	-1.5-39.0

^**a.**^ a score of 3 or less; ^b^. a score between 3 and 6; ^c^. a score of 6 or more

### Behavioral factors

The mean knowledge score of leprosy was 5.7 (95% CI: 5.4–6.0), ranging between 0 and 9 of 126 respondents, 57% had adequate knowledge (score ranging between 6 and 9). Three types of health-seeking behavior were reported as the first action taken when respondents noticed the first signs or symptoms of leprosy. In the first group almost half (57, 45%) applied ‘self-medication’ such as a balm, olive oil, coconut oil, using traditional medicine like “jamu” [herb], galangal or onion, cosmetics, a skin ointment or tinea versicolor medicine, skin care, and giving a compress, or primarily did not seek appropriate healthcare services. For instance, they visited non-qualified practitioners such as nurses or midwives without the capacity to diagnose leprosy. The second group with a total of 48 (38%) respondents, visited an appropriate healthcare service, such as a medical doctor, health center (*Puskesmas*), hospital, dermatologist, and neurologist. The third group consisted of 21 respondents (17%), who initially ignored the leprosy symptoms.

Once respondents sought medication, they visited various kinds of healthcare services. They most often visited a health center (*Puskesmas*) (67, 53%), followed by a general practitioner (36, 28%), dermatologist (16, 13%), hospital (6, 5%), and neurologist (1, 1%). The number of consultations or examinations that respondents had in healthcare services before receiving the leprosy diagnosis ranged from 0 to 8. Fifty respondents (40%) received their leprosy diagnosis on their first visit (Table 1).

### Anticipated Stigma

Most respondents had no family members with leprosy (100, 79%) and had not heard about leprosy before being diagnosed with leprosy (72, 57%). Of all respondents, 48 (38%) thought they would view themselves differently if other people knew that they had leprosy. Most respondents only talked with family or close friends about being diagnosed with leprosy (111, 88%). The mean score of anticipated stigma was 1.9 (95% CI: 1.7–2.2) with a score range from 0 to 4 (Table 1).

### Factors associated with delay

In univariate analysis, both CDD and patient delay as dependent variables, showed a statistically significant correlation in the Pearson correlation test with several independent variables (Additional file 1: Table S2). In multivariate analysis, the following factors remained statistically significant with longer CDD: younger age, being male, found by passive case detection, and not having a family member with leprosy (Table 2). Longer patient delay also showed a statistically significant association in multivariate analysis with younger age, being male, not having a family member with leprosy, and not talking with anybody when they got leprosy as an indication of anticipated stigma in the community (Table 3).

**Table 2:** Linear regression model for the factors related to case detection delay (log_10_)

Variables	Coefficient B	Standard Error Coefficient B	Standardised Coefficient B	t	P-value
Constant	0.748	0.182		4.109	0.000
Age (Years)	-0.011	0.003	-0.246	-3.112	0.002
Sex (0 = Female, 1 = Male)	0.206	0.084	0.194	2.453	0.016
Case detection method (0 = Active, 1 = Passive)	0.313	0.084	0.293	3.704	0.000
A family member had a leprosy patient (0 = Yes, 1 = No)	0.313	0.101	0.247	3.098	0.002

R^2^ = 0.27, F = 10.94, P-value < 0.001

**Table 3:** Linear regression model for the factors related to patient delay (log_10_)

Variables	Coefficient B	Standard Error of Coefficient B	Standardised Coefficient B	t	P-value
Constant	1.308	0.272		4.805	0.000
Age (Years)	-0.012	0.004	-0.266	-3.186	0.002
Sex (0 = Female, 1 = Male)	0.255	0.093	0.227	2.741	0.007
A family member had a leprosy patient (0 = Yes, 1 = No)	0.234	0.112	0.174	2.080	0.040
Talk to anybody when got a leprosy (0 = Yes, 1 = No)	0.463	0.212	0.182	2.184	0.031

R^2^ = 0.19, F = 6.93, P-value < 0.001

## Discussion

The mean CDD, patient delay, and health system delay were 13.0 months, 9.7 months, and 3.2 months, respectively. Factors associated with longer CDD were younger age (below 35 years), being male, being diagnosed through passive case detection, and not having a family member with leprosy. Factors associated with longer patient delay were younger age (below 35 years), being male, not having a family member with leprosy, and anticipated stigma of leprosy in the community. It was not possible to reliably identify factors associated with health system delay.

The mean (overall) CDD in our study (13.0 months) is lower than those reported in India, Bangladesh, Brazil, Nepal, China, Paraguay, Ethiopia, Mozambique, and Tanzania [16, 37–40]. The median CDD in our study (5.5 months) is also lower than the median CDD reported elsewhere in the world, with ranges from 12 to 36 months [16]. The mean patient and health system delay in our study (9.7 and 3.2 months) were also lower than reported in China; Zhang reported a mean patient and health system delay of 24.4 and 25.7 months, respectively, and Chu reported a mean patient and health system delay of 30.1 and 34.3 months, respectively [41, 42]. However, the patient delay in Indonesia is slightly higher than in Nepal (6.5 months) [43]. A worrying finding in our study is that nearly half (48%) of the leprosy patients had a delay of more than 6 months. It is known that nerve damage can become irreversible after 6 months, leading to longstanding disability in those affected [14]. Therefore, although the overall CDD appears lower than in other leprosy endemic countries, it is very important to reduce CDD in a new leprosy patient. This finding should encourage the Indonesian National Leprosy Control Program (NLCP) to implement an intervention programme for the CDD reduction.

We found younger age (less than 35 years) associated with longer CDD, contrary to other studies, where older age was associated with CDD [16]. Incidentally, a long CDD in young people has been reported before; for instance, one report on leprosy in Indonesia referred to a leprosy patient with a long CCD who had already noticed the symptoms of leprosy when he was a teenager [44]. An Indian study mentions a young female with leprosy who said that she was not concerned about seeing the patch that later proved to be the initial sign of leprosy, and therefore did not consult a doctor. Even though her patch was growing, she only used another soap and bathed twice to three times daily [26]. These findings may indicate that young people can have a long CDD because of ignorance about the initial signs and symptoms of leprosy.

Being male was also associated in our study with both a longer overall CDD and patient related CDD. This observation is in line with studies elsewhere in the world [16]. Delay in males is possibly due to difficulties for males to come to health facilities on working days because of loss of income [13, 45].

In our study, leprosy patients with a family member with leprosy had a shorter CDD than those without such a family member. This was also observed in Nepal and in a recent study in Indonesia, where family helped treat relatives with leprosy to avoid the risk of the disease becoming more severe [46, 47]. Also, because families were supported by health staff, they recognised early signs and symptoms of leprosy and knew where to go for treatment, thereby reducing delay [47].

Compared to active case detection, passive case detection was associated with a longer CDD. This confirmed a recent systematic review stating that active case detection produced the shortest detection delay [48]. Active case detection leads to earlier detection and significantly fewer disabilities according to a leprosy expert meeting [15]. An Ethiopian quantitative study showed active case detection and contact tracing to be an important strategy to promote early diagnosis, minimise undiagnosed leprosy cases, and prevent disability [49]. Also, the WHO Global Leprosy Strategy notes that passive case detection is insufficient to interrupt transmission of *M. leprae* [2, 50]. The WHO emphasises integrated active case detection with contact tracing and preventive chemotherapy as one of the strategic pillars of the global leprosy strategy [2, 50].

We found in our study that anticipated stigma of leprosy in the community was associated with patient delay. This is in line with a recent systematic review that reported an association between delay and the fear of isolation in which patients did not disclose their leprosy condition to their community [16]. Several studies linking stigma with delayed case detection of leprosy stated that stigma would make people afraid of leprosy patients. This could cause people afraid of having leprosy to conceal their condition or avoid visiting a healthcare center for medication, thus causing delayed case detection [51–53].

A strength of this study is that the duration of CDD in Indonesia was established with a standardised measurement tool and that relevant factors associated with CDD could be established. The sample size is sufficiently large enough for reliable results that could support the Indonesian NLCP in adjusting its control activities to reduce detection delays. A limitation is that the generalisation of the findings would need to be confirmed in other regions in Indonesia. Since our study design is cross-sectional, we cannot make definitive causal inferences.

We recommend that the Indonesian NLCP improves early case detection through active case finding, including a contact tracing and community awareness campaign through health education, with emphasis on targeting young people, males, and communities with strong leprosy stigma. Examining contacts of new cases in contact tracing is the most cost-effective method for early case detection [15]. Health education is required as an effective intervention to decrease stigma and delays in leprosy detection [54, 55]. One health education method is the “contact intervention” strategy, which invites former leprosy patients to become health educators in their community. It has been a practical and replicable intervention implemented in Indonesia to reduce stigma [56]. Active case detection can be effective if it is undertaken by using mapping to identify high-endemic clusters in an integrated approach to reduce costs and enhance public health benefits, and community engagement to build supportive action in the leprosy programme [50, 57].

## Conclusion

CDD in leprosy should be reduced in Indonesia. The Indonesian NLCP is advised to include an integrated intervention programme combining active case detection with targeted health education to reduce CDD and thereby preventing disabilities in people affected by leprosy.

### Electronic supplementary material

Below is the link to the electronic supplementary material.

Supplementary Material 1

Supplementary Material 2

## Data Availability

All data generated or analysed during this study are included in this published article and its supplementary information files.

## Abbreviations

CDD: Case Detection Delay
DHO: District Health Office
G2D: Grade 2 Disability
MB: Multibacillary
MOH: Ministry of Health
NLCP: National Leprosy Control Program
PB: Paucibacillary
PHO: Provincial Health Office
WHO: World Health Organization
VIF: Variance Inflation Factor

## References

[1] WHO (2020). Global leprosy (Hansen disease) update, 2019: time to step-up prevention initiatives. Wkly Epidemiol Rec.

[2] WHO. Towards zero leprosy. Global leprosy (Hansen’s Disease) strategy 2021–2030. 2021.

[3] WHO (2010). Progress in leprosy control: Indonesia, 1991–2008. Wkly Epidemiol Record = Relevé épidémiologique Hebdomadaire.

[4] WHO (2014). Global leprosy update, 2013; reducing disease burden. Wkly Epidemiol Rec.

[5] Alberts CJ, Smith WCS, Meima A, Wang L, Richardus JH (2011). Potential effect of the world health organization’s 2011–2015 global leprosy strategy on the prevalence of grade 2 disability: a trend analysis. Bull WHO.

[6] WHO (2019). Global leprosy update, 2018: moving towards a leprosyfree world. Wkly Epidemiol Rec.

[7] MOH Republic of Indonesia. Decree of the minister of health of the republic of Indonesia number 11 of 2019 regarding the prevention of leprosy. Jakarta2019. Available from: http://hukor.kemkes.go.id/uploads/produk_hukum/PMK_No__11_Th_2019_ttg_Penanggulangan_Kusta.pdf.

[8] MOH Republic of Indonesia. Indonesia Health Profile. 2019 2020. Available from: https://www.kemkes.go.id/downloads/resources/download/pusdatin/profil-kesehatan-indonesia/Profil-Kesehatan-Indonesia-2019.pdf.

[9] MOH Republic of Indonesia. Indonesia Health Profile 2021. 2022. Available from: https://www.kemkes.go.id/downloads/resources/download/pusdatin/profil-kesehatan-indonesia/Profil-Kesehatan-2021.pdf.

[10] WHO (2022). Global leprosy (Hansen disease) update, 2021: moving towards interruption of transmission–Situation de la lèpre (maladie de Hansen) dans le monde, 2021: vers l’interruption de la transmission. Wkly Epidemiol Record = Relevé épidémiologique Hebdomadaire.

[11] Ogunsumi DO, Lal V, Puchner KP, van Brakel W, Schwienhorst-Stich E-M, Kasang C (2021). Measuring endemicity and burden of leprosy across countries and regions: a systematic review and Delphi survey. PLoS Negl Trop Dis.

[12] Noriega LF, Chiacchio ND, Noriega AF, Pereira GAAM, Vieira ML (2016). Leprosy: ancient disease remains a public health problem nowadays. An Bras Dermatol.

[13] Sabeena J, Bindu RS (2020). Grade 2 disability in Leprosy and its predictors: a 10 year Retrospective Study from Kerala, India. Indian J Lepr.

[14] Nicholls PG, Croft RP, Richardus JH, Withington SG, Smith WCS (2003). Delay in presentation, an indicator for nerve function status at registration and for treatment outcome - the experience of the Bangladesh Acute nerve damage study cohort. Lepr Rev.

[15] Saunderson P (2022). Improving early case detection in leprosy: reports from recent workshops. Lepr Rev.

[16] Dharmawan Y, Fuady A, Korfage I, Richardus JH (2021). Individual and community factors determining delayed leprosy case detection: a systematic review. PLoS Negl Trop Dis.

[17] Von Elm E, Altman DG, Egger M, Pocock SJ, Gøtzsche PC, Vandenbroucke JP (2014). The strengthening the reporting of Observational Studies in Epidemiology (STROBE) Statement: guidelines for reporting observational studies. Int J Surg.

[18] PHO of Central Java Indonesia. Central Java Province Health Profile 2017 2018. Available from: http://dinkesjatengprov.go.id/v2018/dokumen/Profil2017/mobile/index.html#p=1.

[19] PHO of Central Java Indonesia. Central Java Province Health Profile 2021 2022. Available from: https://dinkesjatengprov.go.id/v2018/dokumen/Profil_Kesehatan_2021/mobile/index.html.

[20] Souza CDFd, Tavares DLdC, Tavares CM, Almeida AGCdS, Accioly SMPdS, JPSd P, et al. Physical disabilities due to leprosy in Alagoas State, Northeast Brazil: a temporal and spatial modeling. Volume 52. Revista da Sociedade Brasileira de Medicina Tropical; 2019. 10.1590/0037-8682-0540-2018 31340360

[21] Peters RMH, Van Brakel WH, Lusli M, Damayanti R, Bunders JFG (2017). Cultural validation of a new instrument to measure leprosy-related stigma: the SARI Stigma Scale. Lepr Rev.

[22] Pourhoseingholi MA, Vahedi M, Rahimzadeh M (2013). Sample size calculation in medical studies. Gastroenterol Hepatol bed Bench.

[23] Van ’T, Noordende AT, Lisam S, Ruthindartri P, Sadiq A, Singh V, Arifin M (2021). Leprosy perceptions and knowledge in endemic districts in India and Indonesia: differences and commonalities. PLoS Negl Trop Dis.

[24] Van’t Noordende AT, van Brakel WH (2021). Towards a cross-neglected tropical disease perception study toolkit: a prototype toolkit developed in the field of leprosy. Lepr Rev.

[25] Dharmawan Y, Abqari U, Widjanarko B, Santoso W, Ferdiana A, Richardus JH (2023). The leprosy case detection delay (CDD) questionnaire: indonesian translation, cross-cultural adaptation, and evaluation. Lepr Rev.

[26] Muthuvel T, Govindarajulu S, Isaakidis P, Shewade HD, Rokade V, Singh R (2017). I wasted 3 years, thinking it’s not a problem: patient and Health System Delays in diagnosis of Leprosy in India: a mixed-methods study. PLoS Negl Trop Dis.

[27] Henry M, GalAn N, Teasdale K, Prado R, Amar H, Rays MS (2016). Factors contributing to the Delay in diagnosis and continued transmission of Leprosy in Brazil–An Explorative, quantitative, Questionnaire Based Study. PLoS Negl Trop Dis.

[28] de Bruijne ND, Urgesa K, Aseffa A, Bobosha K, Schoenmakers A, van Wijk R (2022). Development of a questionnaire to determine the case detection delay of leprosy: a mixed-methods cultural validation study. PLoS Negl Trop Dis.

[29] Eberly LE. Multiple linear regression. Topics in Biostatistics. 2007:165 – 87. 10.1007/978-1-59745-530-5_9 18450050

[30] Hambridge T, Coffeng LE, de Vlas SJ, Richardus JH (2023). Establishing a standard method for analysing case detection delay in leprosy using a bayesian modelling approach. Infect Dis Poverty.

[31] van ‘t Noordende AT, Korfage IJ, Lisam S, Arif MA, Kumar A, van Brakel WH (2019). The role of perceptions and knowledge of leprosy in the elimination of leprosy: a baseline study in Fatehpur district, northern India. PLoS Negl Trop Dis.

[32] Tranmer M, Elliot M. Multiple linear regression. The Cathie Marsh Centre for Census and Survey Research (CCSR). 2008;5(5):1–5.

[33] Radboud University. Faculty of social science-Student Information Point (STIP). : Linear Regression: Radboud University; 2023 [cited 2023 22 – 01]. Available from: https://www.ru.nl/socialsciences/stip/facilities-support/facilities/smap/frequently-asked-questions-faq/linear-regression/#linearityisviolated.

[34] Das KR, Imon A (2016). A brief review of tests for normality. Am J Theoretical Appl Stat.

[35] Baalbaki I, Ahmed ZU, Pashtenko VH, Makarem S. Patient satisfaction with healthcare delivery systems. Int J Pharm Healthc Mark. 2008.

[36] Schoenmakers A, Hambridge T, van Wijk R, Kasang C, Richardus JH, Bobosha K (2021). PEP4LEP study protocol: integrated skin screening and SDR-PEP administration for leprosy prevention: comparing the effectiveness and feasibility of a community-based intervention to a health centre-based intervention in Ethiopia, Mozambique and Tanzania. BMJ open.

[37] Mamo E, Bobosha K, Legesse M, Daba F, Debelo K, Leta T (2022). Epidemiological trends of leprosy and case detection delay in East Hararghe Zone, Ethiopia: a baseline survey. Lepr Rev.

[38] Marega A, Hambridge T, Stakteas YP, Schoenmakers A, van Wijk R, Mieras L (2022). Leprosy indicators and diagnosis delay in Mogovolas, Meconta and Murrupula district of Nampula Province, Mozambique: a baseline survey. Lepr Rev.

[39] Mwageni N, Kamara D, Kisonga R, Njako B, Nyakato P, Pegwa A (2022). Leprosy epidemiological trends and diagnosis delay in three districts of Tanzania: a baseline study. Lepr Rev.

[40] Urgesa K, de Bruijne ND, Bobosha K, Seyoum B, Mihret A, Geda B (2022). Prolonged delays in leprosy case detection in a leprosy hot spot setting in Eastern Ethiopia. PLoS Negl Trop Dis.

[41] Zhang F, Chen S, Sun Y, Chu T (2009). Healthcare seeking behaviour and delay in diagnosis of leprosy in a low endemic area of China. Lepr Rev.

[42] Chu T, Liu D, Huai P, Chen X, Han S, Chen S (2020). Comprehensive measures succeeded in improving early detection of leprosy cases in post-elimination era: experience from Shandong province, China. PLoS Negl Trop Dis.

[43] Engelbrektsson UB, Subedi M, Nicholls P (2019). The challenge of health-seeking: recollections of leprosy inpatients in post-elimination Nepal. Lepr Rev.

[44] Pieter Y, Grijsen ML (2022). Picturing health: the burden of leprosy in eastern Indonesia. The Lancet.

[45] Moschioni C, Antunes CMdF, Grossi MAF, Lambertucci JR (2010). Risk factors for physical disability at diagnosis of 19,283 new cases of leprosy. Rev Soc Bras Med Trop.

[46] Robertson LM, Nicholls PG, Butlin R (2000). Delay in presentation and start of treatment in leprosy: experience in an out-patient clinic in Nepal. Lepr Rev.

[47] Nasir A, Yusuf A, Listiawan MY, Makhfudli M (2022). The life experience of leprosy families in maintaining interaction patterns in the family to support healing in leprosy patients in indonesian society. A phenomenological qualitative study. PLoS Negl Trop Dis.

[48] Dharmawan Y, Fuady A, Korfage IJ, Richardus JH (2022). Delayed detection of leprosy cases: a systematic review of healthcare-related factors. PLoS Negl Trop Dis.

[49] Urgesa K, Bobosha K, Seyoum B, Weldegebreal F, Mihret A, Howe R (2021). Evidence for hidden leprosy in a high leprosy-endemic setting, eastern ethiopia: the application of active case-finding and contact screening. PLoS Negl Trop Dis.

[50] Warne G, Mukhier M (2022). Short report for Leprosy Review: ILEP Conference, 2022: active case-finding. Lepr Rev.

[51] Zaw M, Satyanarayana S, Htet KKK, Than K, Aung C (2020). Is Myanmar on the right track after declaring leprosy elimination? Trends in new leprosy cases (2004–2018) and reasons for delay in diagnosis. Lepr Rev.

[52] Sermrittirong S, Van Brakel WH (2014). Stigma in leprosy: concepts, causes and determinants. Lepr Rev.

[53] Marahatta SB, Amatya R, Adhikari S, Giri D, Lama S, Kaehler N (2018). Perceived stigma of leprosy among community members and health care providers in Lalitpur district of Nepal: a qualitative study. PLoS ONE.

[54] Nicholls PG, Ross L, Smith WCS (2006). Promoting early detection in leprosy–A literature review to identify proven and potential interventions addressing patient-related delay. Lepr Rev.

[55] Adhikari B, Kaehler N, Chapman RS, Raut S, Roche P (2014). Factors affecting perceived stigma in leprosy affected persons in western Nepal. PLoS Negl Trop Dis.

[56] Peters RMH, Zweekhorst MBM, Bunders JFG, van Brakel WH (2015). A cluster-randomized controlled intervention study to assess the effect of a contact intervention in reducing leprosy-related stigma in Indonesia. PLoS Negl Trop Dis.

[57] Gillini L, Cooreman E, Pandey B, Bhandari C, Vandelaer J, Rayamajhi R (2018). Implementing the global Leprosy Strategy 2016–2020 in Nepal: Lessons learnt from active case detection campaigns. Lepr Rev.

