# Supplementary material for: Measuring leprosy case detection delay and associated factors in Indonesia: a community-based study
Source: BMC Infect Dis. 2023 Aug 25;23:555. doi: 10.1186/s12879-023-08552-x (PMC10464084; doi:10.1186/s12879-023-08552-x)
Supplement: Supplementary file 1 — Supplementary Material 1 [file 12879_2023_8552_MOESM1_ESM.docx]

**Text S1. Questions related to anticipated stigma of leprosy**

1. Had you heard about leprosy before you were diagnosed with leprosy?
   1. No
   2. Yes
2. When you noticed your first sign, did you think you had leprosy?
   1. No, not at all
   2. Yes, but I was not sure
   3. Yes, I was certain about it
3. What did you do when you noticed your first sign? More answers possible
4. Nothing
5. I asked advice from family or friends
6. I treated it myself
7. I went to the doctor/ health service/drug store
8. Other, namely …
9. Did you expect that other people would think differently about you if they knew about the sign on your body?
   1. No, not at all Score 🡪0
   2. Yes, maybe a little bit Score 🡪1
   3. Yes, absolutely Score 🡪2
10. If yes, why do you expect they would think differently about you? More answers possible (every response option 1 point)
    1. They may think I am unclean (*kush*) Score 🡪1
    2. They may think that I can infect them Score 🡪1
    3. They may think that I may cost them money Score 🡪1
    4. Other, namely… Score 🡪1
11. Did you talk with anybody about the diseases after it was diagnosed?
    1. No Score 🡪2
    2. Yes, only with family or close friends Score 🡪1
    3. Yes, with anybody who showed interest Score 🡪0
