# Supplementary material for: Measuring leprosy case detection delay and associated factors in Indonesia: a community-based study
Source: BMC Infect Dis. 2023 Aug 25;23:555. doi: 10.1186/s12879-023-08552-x (PMC10464084; doi:10.1186/s12879-023-08552-x)
Supplement: Supplementary file 2 — Supplementary Material 2 [file 12879_2023_8552_MOESM2_ESM.doc]

**Table S1. STROBE Statement—Checklist of items that should be included in reports of *cross-sectional studies*

“****Measuring leprosy case detection delay and associated factors in Indonesia: a community-based study.”**

|  | Item No | Recommendation |
| --- | --- | --- |
| **Title and abstract** | 1 | (*a*) Indicate the study’s design with a commonly used term in the title or the abstract  Title, Abstract, |
|  |  | (*b*) Provide in the abstract an informative and balanced summary of what was done and what was found Abstract, |
| Introduction | | |
| Background/rationale | 2 | Explain the scientific background and rationale for the investigation being reported Introduction, all paragraphs; paragraphs 3 and 4 in particular |
| Objectives | 3 | State specific objectives, including any prespecified hypotheses Introduction, final paragraph |
| Methods | | |
| Study design | 4 | Present key elements of study design early in the paper Methods, section ‘study design’ |
| Setting | 5 | Describe the setting, locations, and relevant dates, including periods of recruitment, exposure, follow-up, and data collection Methods, section ‘study area’ |
| Participants | 6 | (*a*) Give the eligibility criteria, and the sources and methods of selection of participants Methods, sections ‘population and sample’ |
| Variables | 7 | Clearly define all outcomes, exposures, predictors, potential confounders, and effect modifiers. Give diagnostic criteria, if applicable Methods, sections ‘study design’ |
| Data sources/ measurement | 8* | For each variable of interest, give sources of data and details of methods of assessment (measurement). Describe comparability of assessment methods if there is more than one group Methods, section ‘study design’ and ‘measurement tool’ |
| Bias | 9 | Describe any efforts to address potential sources of bias Methods, sections ‘data collection and analysis’ |
| Study size | 10 | Explain how the study size was arrived at Methods, section ‘population and sample’ |
| Quantitative variables | 11 | Explain how quantitative variables were handled in the analyses. If applicable, describe which groupings were chosen and why Methods, section ‘data collection and analysis’ |
| Statistical methods | 12 | (*a*) Describe all statistical methods, including those used to control for confounding Methods, section ‘data collection and analysis’ |
|  |  | (*b*) Describe any methods used to examine subgroups and interactions Methods, section ‘data collection and analysis’ |
|  |  | (*c*) Explain how missing data were addressed N/a, there was no missing data |
|  |  | (*d*) If applicable, describe analytical methods taking account of sampling strategy Methods, section ‘data collection and analysis’ |
|  |  | (*e*) Describe any sensitivity analyses Methods, section ‘data collection and analysis’ |
| Results | | |
| Participants | 13* | (a) Report numbers of individuals at each stage of study—eg numbers potentially eligible, examined for eligibility, confirmed eligible, included in the study, completing follow-up, and analysed Results, paragraphs 1 |
|  |  | (b) Give reasons for non-participation at each stage N/a |
|  |  | (c) Consider use of a flow diagram N/a |
| Descriptive data | 14* | (a) Give characteristics of study participants (eg demographic, clinical, social) and information on exposures and potential confounders Results, paragraph 1 |
|  |  | (b) Indicate number of participants with missing data for each variable of interest There were no missing data |
| Outcome data | 15* | Report numbers of outcome events or summary measures Overview in Table 1, details in supporting information files |
| Main results | 16 | (*a*) Give unadjusted estimates and, if applicable, confounder-adjusted estimates and their precision (eg, 95% confidence interval). Make clear which confounders were adjusted for and why they were included Results, Table 1, 2, and 3 |
|  |  | (*b*) Report category boundaries when continuous variables were categorized Results, Table 1 |
|  |  | (*c*) If relevant, consider translating estimates of relative risk into absolute risk for a meaningful time period N/a |
| Other analyses | 17 | Report other analyses done—eg analyses of subgroups and interactions, and sensitivity analyses Results, in **‘**factors associated with delay’, an overview can also be found in in supporting information files. |
| Discussion | | |
| Key results | 18 | Summarise key results with reference to study objectives Discussion, paragraph 1 |
| Limitations | 19 | Discuss limitations of the study, taking into account sources of potential bias or imprecision. Discuss both direction and magnitude of any potential bias Discussion, section ‘strengths and limitations’ |
| Interpretation | 20 | Give a cautious overall interpretation of results considering objectives, limitations, multiplicity of analyses, results from similar studies, and other relevant evidence Discussion, Paragraph 2-8 |
| Generalisability | 21 | Discuss the generalisability (external validity) of the study results Conclusion |
| Other information | | |
| Funding | 22 | Give the source of funding and the role of the funders for the present study and, if applicable, for the original study on which the present article is based  Funding |

*Give information separately for exposed and unexposed groups.

**Note:** An Explanation and Elaboration article discusses each checklist item and gives methodological background and published examples of transparent reporting. The STROBE checklist is best used in conjunction with this article (freely available on the Web sites of PLoS Medicine at http://www.plosmedicine.org/, Annals of Internal Medicine at http://www.annals.org/, and Epidemiology at http://www.epidem.com/). Information on the STROBE Initiative is available at [www.strobe-statement.org](http://www.strobe-statement.org/).

**Table S2. Pearson correlation between the variables included in the study**

|  | Log_10_  Delay | Log_10_  Patient  Delay | A | B | C | D | E | F | G | H | I | J | K | L | M | N | O | P | Q | R | S | T | U | V | W |
| --- | --- | --- | --- | --- | --- | --- | --- | --- | --- | --- | --- | --- | --- | --- | --- | --- | --- | --- | --- | --- | --- | --- | --- | --- | --- |
| **A.** Age (Years) | -0.26** | -0.28** | - |  |  |  |  |  |  |  |  |  |  |  |  |  |  |  |  |  |  |  |  |  |  |
| **B.** Sex (0= Female, 1=Male) | 0.22** | 0.22** | -0.09 | - |  |  |  |  |  |  |  |  |  |  |  |  |  |  |  |  |  |  |  |  |  |
| **C.** Education (Years) | 0.15 | 0.16* | -0.49** | 0.11 | - |  |  |  |  |  |  |  |  |  |  |  |  |  |  |  |  |  |  |  |  |
| **D.** Occupational (0=Unemployed, 1=Employed) | 0.12 | 0.13 | 0.08 | 0.31** | -0.17* | - |  |  |  |  |  |  |  |  |  |  |  |  |  |  |  |  |  |  |  |
| **E.** Marital Status (0=Unmarried, 1=Married) | -0.02 | 0.06 | 0.38* | -0.04 | -0.15* | 0.17** | - |  |  |  |  |  |  |  |  |  |  |  |  |  |  |  |  |  |  |
| **F.** Area of Residence (0=Rural, 1=Urban) | 0.02 | 0.06 | -0.01 | -0.09 | 0.09 | -0.09 | -0.16* | - |  |  |  |  |  |  |  |  |  |  |  |  |  |  |  |  |  |
| **G.** Distance (Km) | 0.05 | 0.07 | -0.12 | -0.02 | 0.11 | -0.15* | -0.05 | 0.01 | - |  |  |  |  |  |  |  |  |  |  |  |  |  |  |  |  |
| **H.** Leprosy Type (0=PB, 1=MB) | 0.10 | 0.04 | 0.04 | 0.00 | -0.05 | 0.14 | -0.03 | 0.19* | 0.05 | - |  |  |  |  |  |  |  |  |  |  |  |  |  |  |  |
| **I.** Case detection method (0=Active, 1=Passive) | 0.36** | 0.18* | -0.09 | 0.10 | 0.12 | 0.07 | -0.03 | 0.12 | 0.05 | 0.14 | - |  |  |  |  |  |  |  |  |  |  |  |  |  |  |
| **J.** Knowledge score (Points) | 0.03 | 0.06 | -0.12 | 0.09 | 0.13 | -0.06 | -0.06 | 0.14 | -0.01 | 0.02 | -0.07 | - |  |  |  |  |  |  |  |  |  |  |  |  |  |
| **K.** Health seeking behaviour to the proper healthcare (0=No, 1=Yes) | -0.01 | -0.17* | 0.07 | -0.07 | 0.01 | -0.05 | -0.02 | 0.04 | -0.12 | -0.12 | -0.02 | -0.09 | - |  |  |  |  |  |  |  |  |  |  |  |  |
| **L.** Health seeking behaviour to the inappropriate healthcare or self medication (0=No, 1=Yes) | 0.11 | 0.19* | -0.19* | 0.01 | 0.11 | 0.02 | -0.08 | 0.05 | 0.19* | 0.16 | 0.13 | 0.04 | -0.72** | - |  |  |  |  |  |  |  |  |  |  |  |
| **M.** Initially ignored the leprosy symptoms (0=No, 1=Yes) | -0.13 | -0.05 | 0.17* | 0.08 | -0.15* | 0.04 | 0.14 | -0.12 | -0.10 | -0.05 | -0.15 | 0.06 | -0.35** | -0.41** | - |  |  |  |  |  |  |  |  |  |  |
| **N.** Healthcare visits: GP (0=No, 1=Yes) | 0.05 | -0.15* | 0.27** | 0.16* | -0.16* | 0.14 | 0.04 | -0.01 | 0.02 | 0.17* | 0.12 | 0.03 | 0.12 | -0.12 | -0.15 | - |  |  |  |  |  |  |  |  |  |
| **O.** Healthcare visits: Dermatologist (0=No, 1=Yes) | 0.04 | -0.02 | -0.16* | -0.10 | 0.23** | -0.24** | -0.08 | 0.11 | 0.02 | 0.06 | -0.06 | 0.23** | 0.04 | -0.01 | -0.04 | -0.24** | - |  |  |  |  |  |  |  |  |
| **P.** Healthcare visits: Health center (0=No, 1=Yes) | -0.08 | 0.14 | -0.12 | -0.03 | 0.00 | 0.06 | 0.12 | -0.13 | -0.04 | -0.25** | -0.08 | -0.21** | -0.18* | 0.12 | 0.09 | -0.67** | -0.41 | - |  |  |  |  |  |  |  |
| **Q.** Healthcare visits: Hospital (0=No, 1=Yes) | 0.08 | 0.06 | -0.09 | -0.06 | -0.02 | -0.00 | -0.19* | 0.09 | 0.04 | 0.11 | 0.02 | 0.07 | 0.06 | 0.02 | -0.10 | -0.14 | -0.09 | -0.24** | - |  |  |  |  |  |  |
| **R.** Number of consultations/ visits to healthcare before leprosy diagnosis (Frequency) | 0.11 | -0.27** | 0.11 | -0.09 | -0.06 | 0.02 | -0.01 | 0.01 | -0.08 | 0.04 | 0.20* | -0.00 | 0.26** | -0.10 | -0.21** | 0.36** | 0.23** | -0.52** | 0.11 | - |  |  |  |  |  |
| **S.** Having family member with leprosy (0=Yes, 1=No) | 0.22** | 0.14 | 0.13 | -0.11 | 0.01 | 0.02 | 0.12 | -0.04 | -0.12 | 0.05 | 0.10 | 0.04 | -0.04 | -0.01 | 0.07 | 0.15* | -0.10 | -0.09 | 0.11 | 0.12 | - |  |  |  |  |
| **T.** Heard about leprosy before being diagnosed with leprosy ((0=No, 1=Yes) | 0.01 | -0.03 | -0.09 | 0.14 | 0.26** | 0.09 | 0.07 | -0.03 | 0.09 | -0.03 | -0.01 | 0.14 | -0.09 | 0.05 | 0.04 | 0.02 | 0.10 | -0.02 | -0.12 | 0.02 | -0.19* | - |  |  |  |
| **U.** Expectation that other people would think differently about them if they knew about the sign on their body (0=No, 1=Yes) | 0.09 | 0.01 | -0.17* | -0.11 | 0.27** | -0.08 | -0.02 | 0.11 | 0.01 | 0.09 | 0.26** | -0.06 | 0.02 | 0.08 | -0.13 | 0.05 | 0.19* | -0.18* | -0.02 | 0.09 | -0.09 | 0.15 | - |  |  |
| **V.** Expectation that other people would think differently about them because a leprosy patient is considered unclean (*kush*) (0=No, 1=Yes) | 0.14 | 0.16* | -0.01 | 0.14 | 0.02 | 0.03 | -0.09 | 0.08 | 0.02 | 0.09 | 0.14 | -0.02 | -0.14 | 0.20* | -0.08 | -0.01 | -0.07 | 0.08 | -0.04 | -0.09 | 0.10 | -0.07 | 0.23** | - |  |
| **W.**Talked with anybody about the disease after being diagnosed with leprosy (0=Yes, 1=No) | 0.16* | 0.21** | -0.09 | -0.06 | 0.15* | 0.16* | 0.14 | -0.07 | -0.00 | -0.18* | 0.02 | -0.07 | 0.06 | -0.13 | 0.10 | -0.06 | 0.03 | 0.06 | -0.05 | -0.07 | 0.11 | 0.03 | 0.13 | -0.04 | - |
| **X.** Stigma Score (Points) | 0.08 | 0.03 | -0.15 | -0.16* | 0.26** | -0.07 | 0.01 | 0.09 | -0.06 | 0.06 | 0.22** | -0.10 | 0.01 | 0.04 | -0.06 | 0.01 | 0.13 | -0.11 | -0.02 | 0.03 | -0.07 | 0.11 | 0.94** | 0.25** | -0.24** |

**Note : * Significant at 5%, ** Significant at 1%**
